# Attitudinal and social factors associated with e-cigarette use among students in formal and non-formal educational settings in Chiang Mai, Thailand

**DOI:** 10.3389/fpubh.2026.1877851

**Published:** 2026-09-24

**Authors:** Pimploy Choradon, Wichuda Jiraporncharoen, Pasiri Singhasiri, Jacqueline E. Hackett, Chaisiri Angkurawaranon, Thanachat Yotruangsri, Apinun Aramrattana, Kanittha Thaikla, Awirut Oon-arom, Thichakorn Tibsri, Nida Buawangpong

**Affiliations:** 1 Department of Family Medicine, Faculty of Medicine, Chiang Mai University, Chiang Mai, Thailand; 2 Global Health and Chronic Conditions Research Center, Chiang Mai University, Chiang Mai, Thailand; 3 Faculty of Medicine, Chiang Mai University, Chiang Mai, Thailand; 4 Department of Health Policy and Management, Johns Hopkins Bloomberg School of Public Health, Baltimore, MD, United States; 5 Research Institute for Health Sciences (RIHES), Chiang Mai University, Chiang Mai, Thailand; 6 Department of Psychiatry, Faculty of Medicine, Chiang Mai University, Chiang Mai, Thailand; 7 Lampang Hospital, Lampang, Thailand

**Keywords:** associated factors, behavior, e-cigarettes, prevalence, secondary school students

## Abstract

**Background:**

E-cigarette use has increased globally and raises concerns about its potential health impacts, particularly among young people, in whom prevalence continues to rise.

**Objective:**

To identify factors associated with its use among students enrolled in formal and non-formal educational institutions in Chiang Mai, Thailand.

**Methods:**

This is a cross-sectional observational study in students, aged 12–25 years old. Data were collected using a structured questionnaire covering sociodemographic characteristics, e-cigarette use behaviors, perceived risk/benefit of e-cigarette use, attitudes toward e-cigarettes, and legal awareness regarding e-cigarettes. Data were analyzed using descriptive statistics and multivariable logistic regression adjusting for age, sex, and school type.

**Results:**

Of the 900 participants, 30.8% had used e-cigarettes and 18.1% reported past-3-month e-cigarette use. Factors significantly associated with past-3-month e-cigarette use included having close contacts who use e-cigarettes (aOR 5.42, 95% CI 3.54–8.30), and favorable attitudes toward e-cigarettes (aOR 7.88 per interquartile-range increase [score 0 to 5], 95% CI 3.97–15.64; overall *p* < 0.001). In contrast, higher perceived risk/benefit of e-cigarette use (aOR 0.53 per interquartile-range increase [score 13 to 18], 95% CI 0.35–0.80; overall *p* = 0.011), and greater legal awareness regarding e-cigarettes (aOR 0.41 per interquartile-range increase [score 10 to 16], 95% CI 0.24–0.71; overall *p* < 0.001) were associated with reduced odds of e-cigarette use.

**Conclusion:**

Social environmental factors, attitudes toward e-cigarettes, perceived risk/benefit of e-cigarettes, and legal awareness were associated with e-cigarette use among young people. These findings may inform evidence-based public health policies and comprehensive prevention strategies aimed at improving risk perception, modifying attitudes toward e-cigarettes, and enhancing knowledge of regulatory frameworks. Future longitudinal intervention studies are needed to evaluate the strategies’ effectiveness.

## 1 Introduction

Electronic cigarettes (e-cigarettes) were originally developed as “less harmful” alternative to conventional combustible cigarettes (1), and were initially marketed as a smoking cessation tool. They use a device that heats a liquid containing nicotine, flavorings, and solvents to generate an inhalable aerosol without burning tobacco (2). E-cigarettes have since evolved considerably in both design and functionality (3). More recently, these products have been used to deliver a variety of substances, including nicotine, which can cause substance-related harms and addiction (4). In 2019, an outbreak of lung injury associated with e-cigarette use in the United States led to the identification of e-cigarette or vaping product use-associated lung injury (EVALI) (5). Evidence suggests that inhalation of e-cigarette aerosols can disrupt pulmonary surfactant, increase alveolar surface tension, and contribute to the development of EVALI, for which no specific treatment currently exists (6). Beyond respiratory effects, e-cigarette use has also been linked to increased risks of cardiovascular disease (7), oral health problems (8), and nicotine dependence comparable to that of conventional cigarettes (9). It may also act as a gateway to the use of other substances (10). Together, these health effects may impose substantial long-term burdens on both individuals and society.

E-cigarette use is particularly prevalent among young people, defined by the United Nations as encompassing adolescents (10–19 years) and youth (15–24 years) (11). In a global systematic review and meta-analysis, the pooled prevalence among secondary school students was 8.8% for ever e-cigarette use and 22.7% for current use. Among high school students, the corresponding prevalences were higher for ever use (33.6%) and 13.0% for current use, highlighting the substantial burden of e-cigarette use in this age group (12). In 2021, the United States’ Centers for Disease Control and Prevention (CDC) reported the highest prevalence of e-cigarette use among individuals aged 18–24 years in the United States (13). A similar pattern was observed in Thailand, where data from the Nation’s Department of Disease Control showed the highest prevalence among those aged 15–24 years (14). Overall, e-cigarette use prevalence among the Thai population rose from 2 to 14 per 10,000 population between 2017 and 2021 (14). More recent nationally representative data from the 2024–2025 National Health Examination Survey further demonstrate that e-cigarette use remains particularly prevalent among younger age groups. Although the overall prevalence of current e-cigarette use was 2.7%, the prevalence was substantially higher among young people aged 10–19 years (5.3%) and young adults aged 20–29 years (8.3%) (15). The variation in prevalence estimates across studies should, however, be interpreted cautiously because estimates may differ according to the age and characteristics of the populations studied, educational settings, and the operational definitions and measurement methods used to assess current e-cigarette use. Several factors may contribute to e-cigarette use among young people, including appealing product designs, marketing exposure, and perceptions that e-cigarettes may assist smoking cessation, despite e-cigarettes not being approved for smoking cessation by the United States Food and Drug Administration (FDA) (16).

Despite strict nationwide bans on the importation and sale of e-cigarettes in Thailand, their use remains prevalent among young people (17). A recent study among Thai upper secondary school students reported a current e-cigarette use prevalence of 23.9%, with parental occupation, attitudes toward e-cigarettes, smoking among peers and family members, and media exposure identified as significant associated factors (18). However, important evidence gaps remain. Previous studies have examined several cognitive and social factors associated with e-cigarette use; however, evidence remains limited regarding how modifiable cognitive factors, particularly perceived risk/benefit of e-cigarettes, personal attitudes, and legal awareness regarding e-cigarettes, interact with social influences on shape e-cigarette use among students across different educational levels and settings. In addition, young people often have limited knowledge of e-cigarette-related legislation and inadequate perceptions of the harms associated with e-cigarette use, while awareness of national tobacco control measures remains low (19). Furthermore, most previous studies have been restricted to upper secondary school students, despite evidence indicating that e-cigarette use may begin as early as primary school (20). These findings underscore the need to investigate e-cigarette use across a broader range of educational levels. Therefore, this study aimed to identify modifiable cognitive and social factors associated with e-cigarette use among Thai students in both formal and informal educational systems.

## 2 Methods

### 2.1 Study design

This study is an observational cross-sectional study.

### 2.2 Study setting

The study was conducted in two educational institutions in Chiang Mai province, Thailand: a government secondary school and a non-formal education center.

### 2.3 Population and sampling

The target population comprised students enrolled in the selected government secondary school and non-formal education center in a district of Chiang Mai, Thailand. Formal education in a government secondary school typically places students in secondary school from ages 12–13 to 17–18 (21), with the upper secondary level corresponding to high school. Non-formal education serves those who paused or left school, allowing them to return and complete their secondary level. Therefore, student ages in this system often vary widely, usually starting above 12 (22). The eligible population comprised all students enrolled in the participating institutions during the study period (*N* = 2,138). Participants were recruited using convenience sampling, with assistance from school personnel in identifying and inviting students who were available during the data collection period. A total of 900 participants were recruited for participation between June and August 2025. Students who were unable to complete the questionnaire, either by their own report or as observed during questionnaire administration, were excluded. No students were excluded on this basis during the study period. A comparison of the demographic characteristics of the source population and the study sample is provided in Supplementary Table S1.

### 2.4 Sample size

To examine factors associated with e-cigarette use, the sample size was further calculated based on the ability to detect an association. Using data from the same previous study, parental e-cigarette use was reported to be associated with increased odds of e-cigarette use among students (adjusted odds ratio (aOR) 1.827 and 95% confidence interval (CI) 1.137–2.938, 23) (50). A power analysis for two proportions was performed to detect an odds ratio of 1.5, yielding a required sample size of 868 participants. Therefore, a total of 900 participants were recruited for this study.

### 2.5 Instruments

The questionnaire used in the present study was adapted from a previously developed and validated Thai questionnaire modified (19) from the Global Health Professions Student Survey (GHPSS), developed by the United States Centers for Disease Control and Prevention (CDC) and the Canadian Public Health Association. The original questionnaire structure and main items were retained, with modifications made to reflect the study objectives and current evidence on e-cigarette use. Specifically, e-cigarette use during the past 3 months (90 days) was used to assess recent use, based on the substance-use assessment approach of the WHO ASSIST, rather than the commonly used 30-day definition of current e-cigarette use, and one additional item assessing whether e-cigarettes can help users quit conventional cigarettes was added based on current evidence regarding their use for smoking cessation (23). The questionnaire includes domains on demographics, prevalence of tobacco use, knowledge and attitudes toward tobacco use, and exposure to secondhand smoke (24). The questionnaire consisted of three sections, as follows:

#### 2.5.1 Section 1: sociodemographic characteristics

This section included age, sex, current educational level, academic performance, average daily allowance, and average monthly household income.

#### 2.5.2 Section 2: e-cigarette use behaviors

Participants were first asked whether they had ever used e-cigarettes in their lifetime. They were then asked whether they had used e-cigarettes within the past 12 months. Those who reported use during the past 12 months were further asked about the frequency of e-cigarette use during the past three months.

#### 2.5.3 Section 3: cognitive and environmental factors associated with e-cigarette use

This section comprised four subdomains: (1) perceived risk/benefit of e-cigarettes (five items), (2) attitudes toward e-cigarette use (three items), (3) legal awareness regarding e-cigarettes (four items), and (4) environmental exposure to e-cigarette use among family members and peers. Environmental exposure was considered present if at least one corresponding item was answered “Yes.” Items in the first three subdomains were measured using a 5-point Likert scale (0 = strongly disagree to 4 = strongly agree). Higher scores in perceived risk/benefit indicate greater awareness of the harmful effects of e-cigarettes (item 1 was reverse coded prior to computing the perceived risk/benefit composite score). Higher scores in legal awareness reflect better understanding of relevant regulations. Higher scores in attitudes toward e-cigarette indicate greater acceptance of e-cigarette use (19).

#### 2.5.4 Reliability of the questionnaire

The internal consistency of the questionnaire was assessed using Cronbach’s alpha. The perceived risk/benefit scale demonstrated Cronbach’s alpha of 0.86, the attitudes toward e-cigarette scale 0.80, and the legal awareness scale 0.89. All three domains were considered to have good reliability, as their Cronbach’s alpha coefficients exceeded the acceptable threshold of 0.70 (25).

#### 2.5.5 Construct validity of the questionnaire

To further examine the internal structure of the questionnaire, exploratory factor analysis (EFA) was conducted for each domain, supporting a unidimensional structure with minimal cross-loading. A three-factor confirmatory factor analysis (CFA) model, specifying perceived risk/benefit, attitude, and legal awareness as distinct latent constructs, demonstrated good fit (CFI = 0.981, TLI = 0.976, RMSEA = 0.055, SRMR = 0.040), with inter-factor correlations (*r* = −0.16 to 0.59) supporting discriminant validity among the three domains. These findings provide empirical support for the proposed three-dimensional structure of the questionnaire.

### 2.6 Data collection and ethics

Data were collected using a structured questionnaire in identical paper-based and online formats via Google Forms, with the same questions, response options, instructions, and item order. Participants chose their preferred questionnaire format. The government secondary school used the online format exclusively (645 participants), while the non-formal education center used paper-based questionnaires for 202 participants and the online format for 53 participants. Paper questionnaires were completed independently, placed in envelopes, and submitted to a research assistant who was not involved in teaching or supervising the students; the responses were subsequently entered into Google Forms. Online questionnaires were completed independently without mandatory items. To protect privacy, no personally identifying information was required, and paper questionnaires were submitted directly to the research assistant rather than school personnel. For participants aged under 20 years, written informed consent was obtained separately from their parents or legal guardians and from the participants themselves before completing the questionnaire. The study was approved by the Institutional Ethics Committee of the Faculty of Medicine, Chiang Mai University (Ethical approval number: 114/2025).

### 2.7 Statistical analysis

Participants were categorized as past-3-month e-cigarette users or non-users based on their reported e-cigarette use within the past 3 months. For descriptive analysis, categorical variables were presented as frequencies and percentages, while continuous variables were summarized using means and standard deviations (SD) and median and interquartile range (IQR). Of 900 enrolled participants, 7 (0.78%) had missing data on age (*n* = 2) or sex (*n* = 5); no participant had missing data for both variables. These participants were excluded via listwise deletion, yielding an analytic sample of 893 participants for all regression models.

To examine associations between baseline characteristics and e-cigarette use, the Chi-square test was applied for categorical variables, and the independent t-test was used for continuous variables. Factors associated with e-cigarette use were further analyzed using multiple logistic regression analysis. The variables including having close contacts who use e-cigarettes, perceived risk/benefit of use, attitudes toward e-cigarettes, and legal awareness regarding e-cigarettes were analyzed as modifiable risk factors. The variables including sex, age and school type were selected as confounders based on prior literature and their theoretical relevance (26–28).

Separate multivariable logistic regression models were fit for each factor associated with past-3-month e-cigarette use. Age and the three continuous psychosocial scale scores (perception toward risk/benefit of e-cigarettes (range 0–20), favorable attitude toward e-cigarettes (range 0–12), and legal awareness regarding e-cigarettes (range 0–16)) showed significant non-linear associations with the outcome and were therefore modeled using restricted cubic splines with four degrees of freedom rather than as linear terms. For each of the three psychosocial scores, the overall association with the outcome was tested with a likelihood ratio test comparing the full model to a model omitting that spline term, and the magnitude of association is summarized as the adjusted odds ratio comparing the 75th to the 25th percentile of the score (OR per interquartile-range increase), with a 95% confidence interval (CI) obtained by the delta method applied to the fitted spline function. Close-contact e-cigarette use (yes/no) was retained as a binary predictor. All separate models were adjusted for age (restricted cubic spline), sex, and school type.

Sensitivity analyses by age group and school type were also performed. Age group was categorized as 18 years or younger versus older than 18 years, and school type was grouped into formal versus non-formal educational settings. In addition, the close-contact variable was further examined in subgroup analyses by relationship type (father, mother, sibling, and friend).

Multicollinearity among the predictors was assessed using the variance inflation factor (VIF). All statistical analyses were performed using STATA version 17 (29). A *p*-value of <0.05 was considered statistically significant.

### 2.8 Definition of sociodemographic variables

Certain variables were categorized to facilitate interpretation. Academic performance was dichotomized by defining “solid academic performance” as a grade point average (GPA) of 2.0 and above. This threshold was based on previous studies demonstrating a significant association between lower academic achievement and e-cigarette use among students in multiple countries, particularly among those with grades corresponding to C (GPA = 2.0), D (GPA = 1.0), and F (GPA = 0) (30).

Household income was also categorized, with “low income” defined as a monthly household income of less than 3,500 Thai Baht (equivalent to ~93 Euro/ $108 USD). This cut-off was based on data from the National Statistical Office of Thailand, Ministry of Digital Economy and Society, which reported that, in 2023, households in the lowest income quintile (quintile 1) had an average monthly income of approximately 3,500 Baht (31). Previous studies have suggested that low household income may be significantly associated with e-cigarette use. This may be explained by increased exposure to targeted marketing strategies among lower-income populations, as well as potential educational inequalities, which may contribute to lower awareness of the risks associated with e-cigarette use (32).

## 3 Results

### 3.1 Participant characteristics

A total of 900 participants consented to complete the questionnaire. Overall, 43.1% were male, 53.9% were female, and 2.9% did not specify their sex. The mean age was 16.5 ± 2.57 years, ranging from 12 to 25 years. Most participants were enrolled in government secondary schools (71.7%), while 28.3% were from non-formal education center. The majority were studying at the upper secondary level (56.0%). Academic performance was generally high, with 36.5% of students reporting a GPA between 3.5 and 4.0. The median monthly household income was 15,000 (IQR 10,000–23,750) Thai Baht [equivalent to US$459 (US$306–US$727)].

### 3.2 Association between e-cigarette use and study variables

Among all participants, 30.8% reported having ever used e-cigarettes and 18.1% reported use within the past 3 months (Table 1). The proportion reporting past-3-month e-cigarette use was 9.0% among students from the government secondary school and 41.2% among students from the non-formal education center. Solid academic performance was higher among students in the non-user group. Having peers or close contacts who used e-cigarettes was associated with higher prevalence of past-3-month e-cigarette use. No statistically significant association was observed between lower-income households and e-cigarette use.

**Table 1:** General characteristics of participants and factors associated.

Characteristic (*N* = 900)	*N*% (CIs)	E-cigarette use		
		Past-3-month e-cigarette use *n* % (CIs)	Non-user *n* % (CIs)	*p*-value
Total	900 (100)	163 (18.1)	737 (81.9)	
Sex				
Male	386 43.1 (39.9–46.4)	88* 54.0 (47.9–63.3)	298 40.4 (36.9–44.0)	0.001
Female	483 53.9 (50.7–57.2)	68* 41.7 (35.5–50.9)	415 56.3 (52.7–59.9)	
Sex not specified	26 2.9 (1.9–4.2)	2* 1.3 (0.3–4.9)	24 3.3 (2.2–4.8)	
Age (mean (CIs))	16.5 (16.33–16.66)	17.4 (17.01–17.84)	16.3 (16.11–16.47)^†^	<0.001
School				
Government secondary school	645 71.7 (68.6–74.6)	58 35.6 (28.6–43.2)	587 79.7 (76.6–82.4)	<0.001
Non-formal educational institution	255 28.3 (25.4–31.4)	105 64.4 (56.8–71.4)	150 20.4 (17.6–23.4)	
Current education level				
Primary school	32 3.6 (2.4–4.9)	8 4.9 (2.5–9.5)	24 3.3 (2.2–4.8)	0.552
Junior high school	364 40.4 (37.3–43.7)	67 41.1 (33.8–48.8)	297 40.3 (36.8–43.9)	
Senior high school	504 56 (52.7–59.2)	88 53.9 (46.3–61.5)	416 56.4 (52.8–59.9)	
Solid academic performance	844 93.8 (91.9–95.2)	139 85.3 (78.9–89.9)	705 95.7 (93.9–96.9)	<0.001
Poor income (less than 3,500 Baht)	57 6.3 (4.8–8.1)	9 5.5 (2.9–10.3)	48 6.5 (4.9–8.5)	0.639
E-cigarette use among close contacts	416 46.2 (42.9–49.5)	125 76.7 (69.6–82.6)	291 39.5 (36.0–43.0)	<0.001

CIs, confidence intervals; * *n* of sex in Past-3-month e-cigarette user was 158 (5 missing data); † *n* of age in Non-user was 735 (2 missing data).

### 3.3 Perceived risk/benefit of e-cigarette, attitudes toward e-cigarettes, and legal awareness regarding e-cigarettes

Table 2 compares the mean scores (CIs) and median (IQR) of cognitive and attitudinal factors between past-3-month e-cigarette users and non-users. Overall, non-users reported a significantly higher perceived risk/benefit of e-cigarettes compared to past-3-month e-cigarette users. Non-users were more likely to recognize that e-cigarettes contain addictive nicotine that could cause neurocognitive damage, and that e-cigarettes are associated with severe inflammatory lung disease, coronary artery disease, and oral health problems. Past-3-month e-cigarette users had a higher mean score regarding the perception that e-cigarette use helps with quitting conventional cigarette smoking compared to non-users. Regarding attitudes, past-3-month e-cigarette users demonstrated significantly more positive attitudes toward e-cigarettes across all surveyed items. Among past-3-month e-cigarette users, the belief that e-cigarette use helps relieve stress was the most frequently reported attitudinal item. Additionally, past-3-month e-cigarette users were more likely to perceive e-cigarette use as a way to enhance personal image and facilitate social interactions with peers compared to their non-using counterparts. In terms of legal awareness, non-users possessed significantly higher legal awareness regarding e-cigarettes. While non-users were more aware that possession and importation of e-cigarettes are illegal and subject to penalties, there was no significant difference between the two groups regarding the knowledge that purchasing or selling e-cigarettes online is illegal.

**Table 2:** Classification 5-point Likert scale for cognitive factors score summation.

Likert scale and questionnaire items Mean (CIs) Median (IQR)	Total	User	Non-user	*p*-value
Perceived risk/benefit of e-cigarettes	14.68 (14.39–14.97) 15 (13–18)	13.73 (13.08–14.38) 14 (12–16)	14.89 (14.57–15.21) 16 (13–18)	0.002
1. E-cigarette use help with quitting conventional cigarette smoking.	2.04 (1.95–2.13) 2 (1–3)	2.28 (2.10–2.46) 2 (2–3)	1.99 (1.89–2.09) 2 (1–3)	0.012
2. E-cigarettes contain nicotine, which is addictive and may cause long-term neurocognitive damage.	3.14 (3.06–3.20) 3 (3–4)	2.83 (2.67–2.98) 3 (2–3)	3.21 (3.13–3.28) 4 (3–4)	<0.001
3. E-cigarette use can lead to severe inflammatory lung disease.	3.21 (3.15–3.28) 3 (3–4)	2.94 (2.80–3.08) 3 (3–4)	3.27 (3.19–3.34) 4 (3–4)	<0.001
4. E-cigarette use increases the risk of coronary artery disease.	3.16 (3.09–3.22) 3 (3–4)	2.91 (2.76–3.06) 3 (3–4)	3.21 (3.14–3.28) 3 (3–4)	<0.001
5. E-cigarette use can cause oral health problems, such as dental caries and tooth discoloration.	3.13 (3.06–3.19) 3 (3–4)	2.76 (2.60–2.92) 3 (2–3)	3.21 (3.14–3.29) 3 (3–4)	<0.001
Attitudes toward e-cigarette	2.92 (2.75–3.10) 3 (0–5)	5.34 (4.96–5.71) 6 (4–7)	2.39 (2.22–2.57) 2 (0–4)	<0.001
6. E-cigarette use enhances personal image and is perceived as fashionable.	0.66 (0.59–0.72) 0 (0–1)	1.29 (1.11–1.46) 1 (0–2)	0.52 (0.46–0.58) 0 (0–1)	<0.001
7. E-cigarette use helps relieve stress.	1.34 (1.27–1.42) 1 (0–2)	2.42 (2.26–2.59) 3 (2–3)	1.11 (1.03–1.18) 1 (0–2)	<0.001
8. E-cigarette use facilitates social interaction with peers.	0.93 (0.86–0.99) 1 (0–2)	1.63 (1.48–1.77) 2 (1–2)	0.77 (0.70–0.84) 0 (0–1)	<0.001
Legal awareness regarding e-cigarettes	11.92 (11.67–12.18) 12 (10–16)	10.91 (10.33–11.48) 12 (8–13)	12.15 (11.87–12.43) 12 (11–16)	<0.001
9. Possession of e-cigarettes is illegal and subject to both imprisonment and fines.	3.03 (2.96–3.09) 3 (3–4)	2.68 (2.52–2.84) 3 (2–3)	3.10 (3.03–3.18) 3 (3–4)	<0.001
10. Importation of e-cigarettes is illegal and subject to both imprisonment and fines.	3.07 (2.99–3.13) 3 (3–4)	2.71 (2.55–2.86) 3 (2–3)	3.15 (3.07–3.22) 3 (3–4)	<0.001
11. The purchase and sale of e-cigarettes, including online transactions, are illegal.	2.72 (2.63–2.80) 3 (2–4)	2.59 (2.43–2.76) 3 (2–3)	2.74 (2.65–2.84) 3 (2–4)	0.182
12. Using e-cigarettes in smoke-free areas is illegal and subject to a monetary fine.	3.11 (3.04–3.18) 3 (3–4)	2.93 (2.76–3.09) 3 (2–4)	3.15 (3.08–3.23) 3 (3–4)	0.013

CIs, confidence intervals; IQR, interquartile range.

### 3.4 Factors associated with past-3-month e-cigarette use

Of 900 enrolled participants, 893 were included in the multivariable analyses after excluding those with missing age or sex (*n* = 7). Close-contact e-cigarette use was strongly associated with past-3-month e-cigarette use (adjusted OR 5.42, 95% CI 3.54–8.30). Because age and the three psychosocial scale scores showed significant non-linear associations with past-3-month e-cigarette use (Table 3), these were modeled using restricted cubic splines rather than linear terms. Perception toward risk/benefit of e-cigarettes was inversely associated with past-3-month e-cigarette use (OR 0.53 per interquartile-range increase [score 13 → 18], 95% CI 0.35–0.80; overall *p* = 0.011). Favorable attitude toward e-cigarettes showed a strong positive, non-linear association, with risk accelerating sharply at higher scores (OR 7.88 per interquartile-range increase [score 0 → 5], 95% CI 3.97–15.64; overall *p* < 0.001). Legal awareness regarding e-cigarettes was inversely associated with past-3-month e-cigarette use (OR 0.41 per interquartile-range increase [score 10 → 16], 95% CI 0.24–0.71; overall *p* < 0.001).

**Table 3:** Complete regression results for all four models (*n* = 893).

Covariate	Category (ref.)	aOR	95% CI	*p*-value
E-cigarette use among close contacts	Yes vs. No	5.42	3.54–8.30	<0.001
Perception toward risk/benefit (score 0–20)	OR per IQR (13 → 18)	0.53	0.35–0.80	0.011
Favorable attitude (score 0–12)	OR per IQR (0 → 5)	7.88	3.97–15.64	<0.001
Legal awareness of e-cig laws (score 0–16)	OR per IQR (10 → 16)	0.41	0.24–0.71	<0.001

Adjusted for age (restricted cubic spline), sex, and school type.

### 3.5 Subgroup and exploratory analyses

The sensitivity analyses by age and school type are also shown in Supplementary Table S2. Both school types and both age groups show the same direction and broad pattern of association as the main analysis (Table 3). Additional analyses disaggregating close-contact e-cigarette use by type of close contact are presented in Supplementary Table S3. Positive associations with past-3-month e-cigarette use were observed across the different types of close contacts, with the largest point estimate observed for friends (aOR 5.30).

## 4 Discussion

The findings revealed that 30.8% of participants had ever used e-cigarettes, while 18.1% were past-3-month e-cigarette users (having used within the last 3 months). Having close contacts who use e-cigarettes was also associated with higher odds of past-3-month e-cigarette use among students. In terms of cognitive factors, higher levels of perceived risk/benefit of e-cigarette use and greater legal awareness regarding e-cigarettes were associated with lower odds of e-cigarette use. Conversely, favorable attitudes toward e-cigarettes were strongly associated with past-3-month e-cigarette use. The restricted cubic spline analysis further showed that this association was non-linear, with the predicted probability of e-cigarette use increasing more markedly at higher attitude scores, particularly above the sample median, rather than increasing at a constant rate across the scale. This pattern may suggest a threshold-like association, whereby favorable attitudes become more strongly associated with e-cigarette use at higher levels of favorable attitudes. These findings align with established behavioral theory suggesting that e-cigarette use among young people is driven by an interaction between attitudes, perceived norms, and knowledge. While legal awareness and perceived health risks were associated with lower odds of e-cigarette use, these appear to be outweighed in some cases by favorable attitudes and strong peer influences, indicating that social norms and affective beliefs may be more closely associated with e-cigarette use. These findings may be interpreted through the lens of the Theory of Planned Behavior (TPB), which posits that behavioral intentions are shaped by attitudes, subjective norms, and perceived behavioral control (33, 34). However, the present study did not assess all major TPB constructs, particularly behavioral intentions and perceived behavioral control, nor did it test a mediation or structural model. TPB is therefore offered here as a plausible interpretive framework rather than as a theory confirmed by this study.

The prevalence of both ever and past-3-month e-cigarette use among secondary school students in this study is concerning when compared with previous findings. A global estimate in 2024 reported the prevalence of having ever used e-cigarettes among students across all educational levels at 22.7%, and current use at 12.9%, with variations across geographic regions (12). In Southeast Asia, the prevalence of having ever used e-cigarettes was 27.4%, with current use at 11% (35). Regulatory factors may also play a critical role. Singapore, for instance, has implemented comprehensive bans on e-cigarettes with strict enforcement activities, and no prevalence data have been reported (36). In Thailand, national data from 2022 indicated a prevalence of e-cigarette use of 1.2% (37) whereas a 2025 study among students in one Thai province reported a current-use prevalence of 23.9% (18), which was comparable to the findings of the present study. However, these estimates should be interpreted cautiously because they were derived from different populations, geographic settings, sampling strategies, and potentially different definitions and measurement periods of current e-cigarette use. Therefore, these findings do not provide sufficient evidence of a temporal increase in e-cigarette use. The relatively high prevalence observed among students in the present study, despite existing legal restrictions on the importation, sale, possession, and public use of e-cigarettes in Thailand, may reflect regional or contextual differences and warrants further investigation. It is also important to note that past-3-month e-cigarette use in this study was defined as e-cigarette use within the past 90 days (3 months), based on the substance-use assessment approach of the World Health Organization’s Alcohol, Smoking and Substance Involvement Screening Test (ASSIST) instrument. This definition differs from the 30-day assessment period commonly used to define current e-cigarette use in previous studies (19, 38). The longer assessment period may partly explain differences in prevalence compared with studies using a 30-day assessment period; therefore, direct comparisons should be made with caution. Age of past-3-month e-cigarette users was higher than non-users. This finding is consistent with data from the 2023 National Health Interview Survey in the United States which reported a higher prevalence of e-cigarette use among individuals aged 21–24 years (15.5%) compared with those aged 18–20 years (10.3%) (39). Prior reviews also indicate that age, sex, socioeconomic status, and academic performance are well-established, cross-cutting correlates of young people’s substance use. For e-cigarettes specifically, a 75-country analysis of Global Youth Tobacco Survey data found that older age, male sex, and higher country income were associated with greater odds of current use (40). This pattern may be explained by the age distribution of the study population (12–25 years), which overlaps with both of the age groups reported in the aforementioned data. It is plausible that as young people grow older and approach adulthood, they gain greater financial autonomy, expanded access to e-cigarette supplies through social networks or informal markets, and independence in decision-making, which may increase the likelihood of engaging in risk behaviors. This is supported by developmental theories suggesting increased risk-taking during late adolescence, including the dual systems model, which explains that such behavior arises from an imbalance between a highly reactive socioemotional system and an immature cognitive control system. This developmental mismatch may be associated with greater engagement in risk behaviors, such as e-cigarette use (41).

In the multiple logistic regression analysis, several factors associated with e-cigarette use were consistent with previous research, particularly environmental factors such as having close contacts who use e-cigarettes. A study conducted in Thailand using student survey data from 2019 found that environmental factors, including parental and peer e-cigarette use, were significantly associated with e-cigarette use among young people (20). Parental smoking behavior, whether involving e-cigarettes or conventional cigarettes, has been shown to significantly associated with young people’s likelihood of e-cigarette use. This may be partly explained by parental attitudes. Young people whose parents hold favorable views toward e-cigarette use are more likely to adopt similar behaviors. Conversely, open communication between parents and children regarding smoking has been associated with reduced e-cigarette use among young people (42). Peer influence has also been consistently identified as a significant factor associated with both ever and past-3-month e-cigarette use, particularly among individuals aged 18 years (43). These findings are consistent with the concept of social modeling and peer influence, which are widely recognized as key determinants of health-related behaviors in young people (44). Meta-analytic evidence further indicates that e-cigarette use among close contacts is strongly associated with e-cigarette initiation and continued use among young people (45). Taken together, these findings suggest that the social environment may be an important correlate of e-cigarette use in this population and that future prevention efforts may benefit from addressing peer and family influences alongside individual-level factors (43).

Cognitive factors were also associated with use, consistent with prior evidence. Lower perceived health risks and more favorable attitudes toward e-cigarette use, particularly beliefs that e-cigarettes promote relaxation and facilitate social interaction, were significantly associated with past-3-month e-cigarette use (19). These findings are consistent with a study conducted among university students in Palestine in 2023 (46), which reported that more than 60% of participants had inadequate knowledge, particularly among current e-cigarette users. Notably, 24.8% expressed disagreement with bans on e-cigarette use in public places. Additionally, only 40.2% of participants demonstrated appropriate attitudes toward e-cigarette use, which was associated with a lower likelihood of use (46). This aligns with the role of legal awareness regarding e-cigarettes. In the present study, past-3-month e-cigarette users had significantly lower levels of accurate knowledge regarding legal regulations of e-cigarettes compared with non-users. One possible explanation is that awareness of Thailand’s strict regulations may be associated with greater recognition of e-cigarettes as prohibited substances and greater concern about their legal consequences. Evidence from the United States, where regulations are comparatively less stringent but include measures such as taxation, packaging requirements, youth access restrictions, and licensing requirements for retailers, suggests that each of these regulatory approaches is associated with reductions in both e-cigarette use and initiation of smoking behaviors (47).

Favorable attitudes toward e-cigarettes were associated with e-cigarette use, consistent with literature identifying attitudes as one of the proximal predictors of substance use behavior (45, 48, 49). While users typically maintain positive associations, viewing e-cigarette use as a tool for stress management, social belonging, or focus, non-users undergo a cognitive shift toward prioritizing negative outcomes like health risks, financial costs, and the burden of addiction. This discrepancy may also be interpreted through the TPB, which posits that behavior is driven by intentions – shaped not just by knowledge, but by personal attitudes, perceived social pressure, and self-efficacy (33, 34). The strong association between attitudes and e-cigarette use suggests that prevention strategies may need to address affective beliefs and social meanings attached to e-cigarette use, rather than focusing solely on providing factual information. Peer-educator programs involving influential students to promote accurate information about e-cigarettes, together with family education, may therefore be worth considering as potential prevention strategies. These associations are consistent with well-established correlates of substance use among young people, including tobacco, alcohol, and cannabis, for which attitudes, perceived risk, and knowledge have been associated with substance use across different substances (48, 49). However, our study extends this evidence to younger people than those typically examined in prior literature, highlighting that these factors are associated with e-cigarette use at younger ages than previously documented.

The disconnect between strict national regulations and high observed prevalence suggests that regulatory presence alone is insufficient without effective enforcement and complementary behavioral interventions. Evidence from cross-national studies indicates that regulatory frameworks are most effective when paired with enforcement mechanisms, retail control, and public awareness campaigns (47). In the Thai context, the persistence of e-cigarette use despite existing legal restrictions warrants further investigation of factors such as enforcement, informal or online markets, and risk perceptions among young people. However, these factors were not directly assessed in the present study. Further research is needed to examine their associations with e-cigarette use and to inform comprehensive public health strategies. Although legal awareness regarding e-cigarettes was associated with reduced use, the overall effect size was modest, suggesting that awareness alone may not be sufficient to drive behavior change. This may reflect the well-documented “knowledge–behavior gap” in public health, whereby awareness of risks or regulations does not necessarily translate into behavioral modification (32). This gap may be particularly pronounced among young people, for whom social and emotional factors may be more closely associated with e-cigarette use than cognitive risk assessments. Future research should prioritize longitudinal designs to better establish causal pathways between cognitive, social, and behavioral factors. In addition, mixed-methods approaches incorporating qualitative data may provide deeper insight into how young people interpret risk, legality, and social norms surrounding e-cigarette use. Given the observed association between close-contact e-cigarette use and participants’ e-cigarette use, social network analysis may be a useful approach for further understanding social patterns of e-cigarette use and identifying potential targets for future intervention research among young people.

The strengths of this study include a relatively large sample size and the use of multiple logistic regression analysis to control for important confounding variables, thereby enhancing the robustness of the findings. However, this study has several limitations. First, due to a cross-sectional design, causal relationships cannot be definitively established. Second, the data were based on self-reported responses from students, which may be subject to reporting bias, particularly due to concerns about providing truthful answers. Third, participants were recruited by convenience sampling from only two educational institutions in a single district of Chiang Mai Province. The study population included students from formal and non-formal educational settings and comprised participants aged 12–25 years, including 32 students classified at the primary school level. Therefore, the sample should not be considered representative of secondary school students in Thailand as a whole, and the findings may have limited generalizability to students in other educational settings or geographic areas. Fourth, although EFA and CFA provided empirical support for the proposed three-factor structure and discriminant validity of the scales, the questionnaire was adapted for this study, and its measurement properties may not be fully generalizable to other populations or educational settings. Fifth, although multiple logistic regression was used to adjust for several important potential confounders, residual confounding cannot be excluded because some potentially relevant factors may not have been measured or fully captured in the model. In the primary analysis, close-contact e-cigarette use was treated as an aggregated variable, which may have obscured differences across relationship types. However, additional subgroup analyses were conducted to examine these associations separately by relationship type (father, mother, sibling, and friend), as presented in Supplementary Table S3. Sixth, the use of multiple item-level comparisons may have increased the risk of type I error due to multiple testing; therefore, findings from individual item-level analyses should be interpreted cautiously. Seventh, the use of a 90-day definition for past-3-month e-cigarette use, while justified, limits direct comparability with studies using the standard 30-day measure. This may result in higher prevalence estimates relative to studies using a 30-day assessment period.

## 5 Conclusion

This study identified several factors associated with e-cigarette use, including having close contacts who use e-cigarettes, perceived risk/benefit of e-cigarette use, attitudes toward e-cigarettes, and legal awareness regarding e-cigarettes. These factors may represent relevant targets for future longitudinal and intervention research, particularly research aimed at enhancing awareness of health risks, correcting misconceptions, reducing favorable attitudes toward e-cigarette use, and understanding the role of social influences among young people.

## Data Availability

The data analyzed in this study is subject to the following licenses/restrictions: the data of this study can be obtained from the corresponding author upon reasonable request and subsequent review by the author. Requests to access these datasets should be directed to nidalooknum@gmail.com.
