# Supplementary material for: Attitudinal and social factors associated with e-cigarette use among students in formal and non-formal educational settings in Chiang Mai, Thailand
Source: Front Public Health. 2026 Sep 24;14:1877851. doi: 10.3389/fpubh.2026.1877851 (PMC13645515; doi:10.3389/fpubh.2026.1877851)
Supplement: Supplementary file 1 [file Table_1.DOCX]

**Supplementary Table S1. Comparison of the source population and the study sample**

| **Characteristic** | **Source population (N = 2,138)** | **Study sample (N = 900)** |
| --- | --- | --- |
| Male | 1,007 (47.1%) | 386 (42.9%) |
| Female | 1,131 (52.9%) | 483 (53.7%) |
| Gender not specified | - | 26 (2.9%) * |
| School |  |  |
| Government secondary school | 1,595 (74.6%) | 645 (71.7%) |
| Non-formal educational institution | 543 (25.4%) | 255 (28.3%) |
| Current education level |  |  |
| Primary school | 83 (3.9%) | 32 (3.6%) |
| Junior high school | 1,044 (48.8%) | 364 (40.4%) |
| Senior high school | 1,011 (47.3%) | 504 (56%) |

* n of sex was 895 (5 missing data)
