# Supplementary material for: Attitudinal and social factors associated with e-cigarette use among students in formal and non-formal educational settings in Chiang Mai, Thailand
Source: Front Public Health. 2026 Sep 24;14:1877851. doi: 10.3389/fpubh.2026.1877851 (PMC13645515; doi:10.3389/fpubh.2026.1877851)
Supplement: Supplementary file 2 [file Table_2.DOCX]

**Supplementary Table S2** Subgroup analysis by age and school type

| **Covariate** | **Category (ref.)** | **aOR** | **95% CI** | **p-value** |
| --- | --- | --- | --- | --- |
| **Age ≤ 18 years (n = 769)**  Adjusted for sex and school type. | | | | |
| E-cigarette use among close contacts | Yes vs No | 5.55 | 3.38-9.13 | <0.001 |
| Perception toward risk/benefit (score 0-20) | OR per IQR (13→18) | 0.80 | 0.63-1.01 | 0.064 |
| Favorable attitude (score 0-12) | OR per IQR (0→5) | 6.29 | 4.04-9.80 | <0.001 |
| Legal awareness of e-cig laws (score 0-16) | OR per IQR (10→16) | 0.72 | 0.53-0.99 | 0.043 |
| **Age > 18 years (n = 124)**  Adjusted for sex and school type. | | | | |
| E-cigarette use among close contacts | Yes vs No | 4.09 | 1.73-9.69 | 0.001 |
| Perception toward risk/benefit (score 0-20) | OR per IQR (13→18) | 0.47 | 0.27-0.82 | 0.008 |
| Favorable attitude (score 0-12) | OR per IQR (0→5) | 10.46 | 3.69-29.64 | <0.001 |
| Legal awareness of e-cig laws (score 0-16) | OR per IQR (10→16) | 0.38 | 0.19-0.74 | 0.005 |
| **Formal educational setting (n = 645)**  Adjusted for age (restricted cubic spline) and sex. | | | | |
| E-cigarette use among close contacts | Yes vs No | 7.04 | 3.54-13.97 | <0.001 |
| Perception toward risk/benefit (score 0-20) | OR per IQR (13→18) | 0.79 | 0.61-1.03 | 0.085 |
| Favorable attitude (score 0-12) | OR per IQR (0→5) | 6.66 | 3.88-11.43 | <0.001 |
| Legal awareness of e-cig laws (score 0-16) | OR per IQR (10→16) | 0.65 | 0.45-0.94 | 0.023 |
| **Non-formal educational setting (n = 248)**  Adjusted for age (restricted cubic spline) and sex. | | | | |
| E-cigarette use among close contacts | Yes vs No | 4.53 | 2.54-8.06 | <0.001 |
| Perception toward risk/benefit (score 0-20) | OR per IQR (13→18) | 0.66 | 0.45-0.95 | 0.025 |
| Favorable attitude (score 0-12) | OR per IQR (0→5) | 7.65 | 4.05-14.44 | <0.001 |
| Legal awareness of e-cig laws (score 0-16) | OR per IQR (10→16) | 0.64 | 0.42-0.98 | 0.041 |
