# Supplementary material for: Attitudinal and social factors associated with e-cigarette use among students in formal and non-formal educational settings in Chiang Mai, Thailand
Source: Front Public Health. 2026 Sep 24;14:1877851. doi: 10.3389/fpubh.2026.1877851 (PMC13645515; doi:10.3389/fpubh.2026.1877851)
Supplement: Supplementary file 3 [file Table_3.DOCX]

**Supplementary table S3** Close contact who uses e-cigarettes

| **Covariate** | **Category (ref.)** | **aOR** | **95% CI** | **p-value** |
| --- | --- | --- | --- | --- |
| Father uses e-cigarettes (n = 847) | Yes vs No | 2.33 | 1.27-4.28 | 0.006 |
| Mother uses e-cigarettes (n = 842) | Yes vs No | 2.81 | 1.15-6.90 | 0.024 |
| Sibling uses e-cigarettes (n = 848) | Yes vs No | 3.70 | 2.20-6.22 | <0.001 |
| Friend uses e-cigarettes (n = 860) | Yes vs No | 5.30 | 3.49-8.05 | <0.001 |

Adjusted for age (restricted cubic spline), sex, and school type.
